# A motion-classification strategy based on sEMG-EEG signal combination for upper-limb amputees

**DOI:** 10.1186/s12984-016-0212-z

**Published:** 2017-01-07

**Authors:** Xiangxin Li, Oluwarotimi Williams Samuel, Xu Zhang, Hui Wang, Peng Fang, Guanglin Li

**Affiliations:** 1 Chinese Academy of Sciences (CAS) Key Laboratory of Human-Machine Intelligence-Synergy Systems, Shenzhen Institutes of Advanced Technology, Shenzhen, 518055 China; 2 Shenzhen College of Advanced Technology, University of Chinese Academy of Sciences, Shenzhen, 518055 China; 3 Department of Biology, South University of Science and Technology of China, Shenzhen, 518055 China

**Keywords:** Amputee, EEG, Hybrid interface, Motion classification, Multifunctional prosthesis, Pattern recognition, Rehabilitation, sEMG, Signal Fusion

## Abstract

**Background:**

Most of the modern motorized prostheses are controlled with the surface electromyography (sEMG) recorded on the residual muscles of amputated limbs. However, the residual muscles are usually limited, especially after above-elbow amputations, which would not provide enough sEMG for the control of prostheses with multiple degrees of freedom. Signal fusion is a possible approach to solve the problem of insufficient control commands, where some non-EMG signals are combined with sEMG signals to provide sufficient information for motion intension decoding. In this study, a motion-classification method that combines sEMG and electroencephalography (EEG) signals were proposed and investigated, in order to improve the control performance of upper-limb prostheses.

**Methods:**

Four transhumeral amputees without any form of neurological disease were recruited in the experiments. Five motion classes including hand-open, hand-close, wrist-pronation, wrist-supination, and no-movement were specified. During the motion performances, sEMG and EEG signals were simultaneously acquired from the skin surface and scalp of the amputees, respectively. The two types of signals were independently preprocessed and then combined as a parallel control input. Four time-domain features were extracted and fed into a classifier trained by the Linear Discriminant Analysis (LDA) algorithm for motion recognition. In addition, channel selections were performed by using the Sequential Forward Selection (SFS) algorithm to optimize the performance of the proposed method.

**Results:**

The classification performance achieved by the fusion of sEMG and EEG signals was significantly better than that obtained by single signal source of either sEMG or EEG. An increment of more than 14% in classification accuracy was achieved when using a combination of 32-channel sEMG and 64-channel EEG. Furthermore, based on the SFS algorithm, two optimized electrode arrangements (*10-channel sEMG + 10-channel EEG*, *10-channel sEMG + 20-channel EEG*) were obtained with classification accuracies of 84.2 and 87.0%, respectively, which were about 7.2 and 10% higher than the accuracy by using only 32-channel sEMG input.

**Conclusions:**

This study demonstrated the feasibility of fusing sEMG and EEG signals towards improving motion classification accuracy for above-elbow amputees, which might enhance the control performances of multifunctional myoelectric prostheses in clinical application.

**Trial registration:**

The study was approved by the ethics committee of *Institutional Review Board of Shenzhen Institutes of Advanced Technology*, and the reference number is *SIAT-IRB-150515-H0077.*

## Background

Multifunctional prostheses are commonly used by upper-limb amputees to restore their lost motion functions. Surface electromyography (sEMG) is a kind of neural signal that contains motor commands and can be non-invasively extracted on the muscle surface of residual limbs. Due to its relative ease of acquisition and abundant content of neural information, sEMG plays an important role in the control of modern motorized prostheses [1–4] and rehabilitation robotics [4–6]. In actually applications, however, the residual muscles after amputations are usually limited, especially in the case of above-elbow amputations. Thus, there exists a dilemma that the less the residual muscles are available for prosthesis control, the more the joint movements would be expected. As a result, multifunctional myoelectric prostheses for above-elbow amputees are still seldom seen on the market [7, 8].

Electroencephalography (EEG) is another kind of neural signal that contains the information related to mental activities of brain but is independent of amputation conditions [9]. Several efforts have been exploited to apply EEG as a brain-computer interface (BCI) for possible applications: Hochberg et al. applied EEG to control robotic arms to perform hand movements for paralyzed subjects, by decoding EEG signals recorded with microelectrode array implanted in the motor cortex [10]; Gernot et al. developed a non-invasive BCI system based on the steady-state visual evoked potentials (SSVEPs) to control a prosthetic hand, where a varied classification accuracy between 44 and 88% was obtained on able-bodied subjects [11]. However, EEG is usually limited for clinical use due to either the biocompatibility of implantable electrode and signal instability for long term application in an invasive way or the low information transmission rate, low spatial resolution, and high signal variability in a non-invasive way [1, 12].

Multiple-source signal fusion is a possible solution for the problem of insufficient information in prosthesis control [13, 14], where some non-EMG signals are combined to sEMG signals to realize a more precise extraction of motor commands. A method based on the combination of sEMG and near-infrared (NIR) signals was reported [15], where hand gestures could be identified by adjoining two or more sEMG/NIR sensors. However, the NIR signals are associated to limb movements and thus still depend on the amputation condition. Another alternative non-EMG signal for prosthesis control may be the human voice, which is irrelevant to the limb movements and independent of amputation levels. Some previous works have demonstrated the feasibility of using speech as an additional input to realize a flexible control of multifunctional myoelectric prostheses [16]. However, the human speech is non-task-related and amputees would feel awkward to pronounce commands during prosthesis operation.

In this work, a non-invasive hybrid method was developed, which coherently combined sEMG and EEG signals as a parallel input to classify the upper-limb motions for above-elbow amputees. The proposed method was additionally optimized by channel selections in order to enhance the classification performance with a decreased number of electrodes.

## Methods

### Subjects

Four male transhumeral amputees (TH1, TH2, TH3, and TH4) were recruited in the experiments, and the demographic information is described in Table 1. Comparing the stumps (measured from shoulder blade downwards) with the intact side opposite to the amputation side of each amputee, the residual limb length ranges around from 1/3 to 1/2 of the intact limb length. The experimental protocol was approved by the Institutional Review Board of Shenzhen Institutes of Advanced Technology, Chinese Academy of Sciences. All subjects have provided written permission for publication of photographs for scientific and educational purposes.

**Table 1:** Demographic information of subjects

Subject	Age (years)	Amputation side	Stump length^a^ (cm)	Time since amputation (years)
TH1	49	Left	20	3
TH2	46	Left	25	9
TH3	35	Right	27	5
TH4	36	Right	30	7

^a^Stump length was measured from shoulder blade downwards

### Signal acquisition and processing

**Fig. 1:**
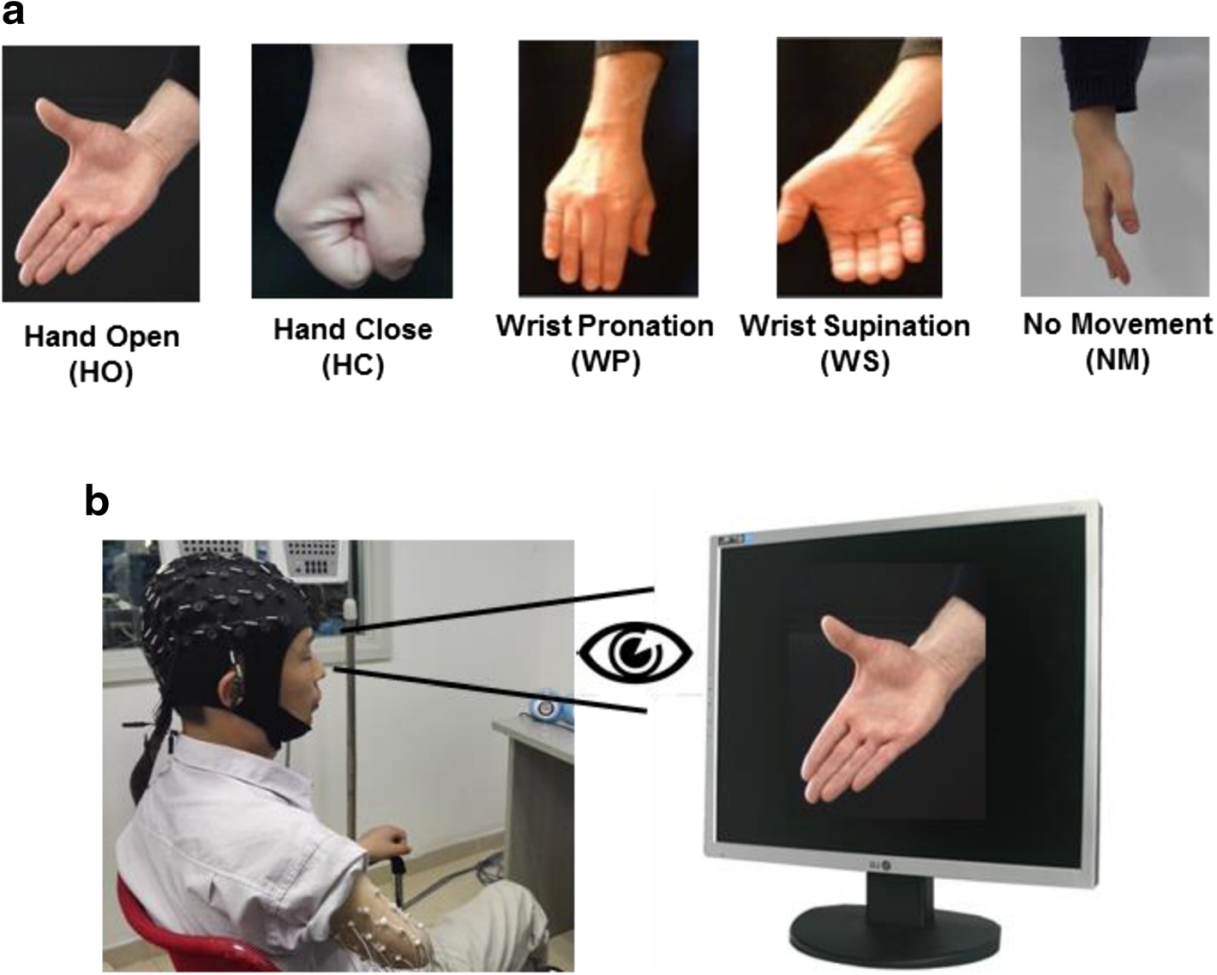
**a** Motion classes involved in the study; **b** A subject performed the target movement (hand open) displayed on the screen by the motion picture

Five motion classes of hand open (HO), hand close (HC), wrist pronation (WP), wrist supination (WS), and no movement (NM) were tested, as shown in Fig. 1a. All subjects were clearly informed about the experiments and trained for a few minutes to get familiar with the procedure. During the experiment, a computer screen was placed in the front of subjects and they were asked to watch the screen for the instructions to do a movement. Each of all the motion classes involved in the study would be displayed with a motion picture on the screen with a random order as a target movement. When a picture of the target movement appeared on the screen, as presented in Fig. 1b, the subjects would promptly perform it. And then when the target motion picture disappeared, the subject stopped doing the movement. To avoid muscle and mental fatigue, the five motion classes were randomly performed with a comfortable force level determined by the subjects. Each motion was hold for 5 s and a rest of 5 s (i.e. NM) was scheduled between two neighboring motions. Five data recording sessions were specified to each subject, where each session consisted of 40 active motions (each of HO, HC, WP, and WS appeared 10 times randomly) and 40 repetition of NM.

**Fig. 2:**
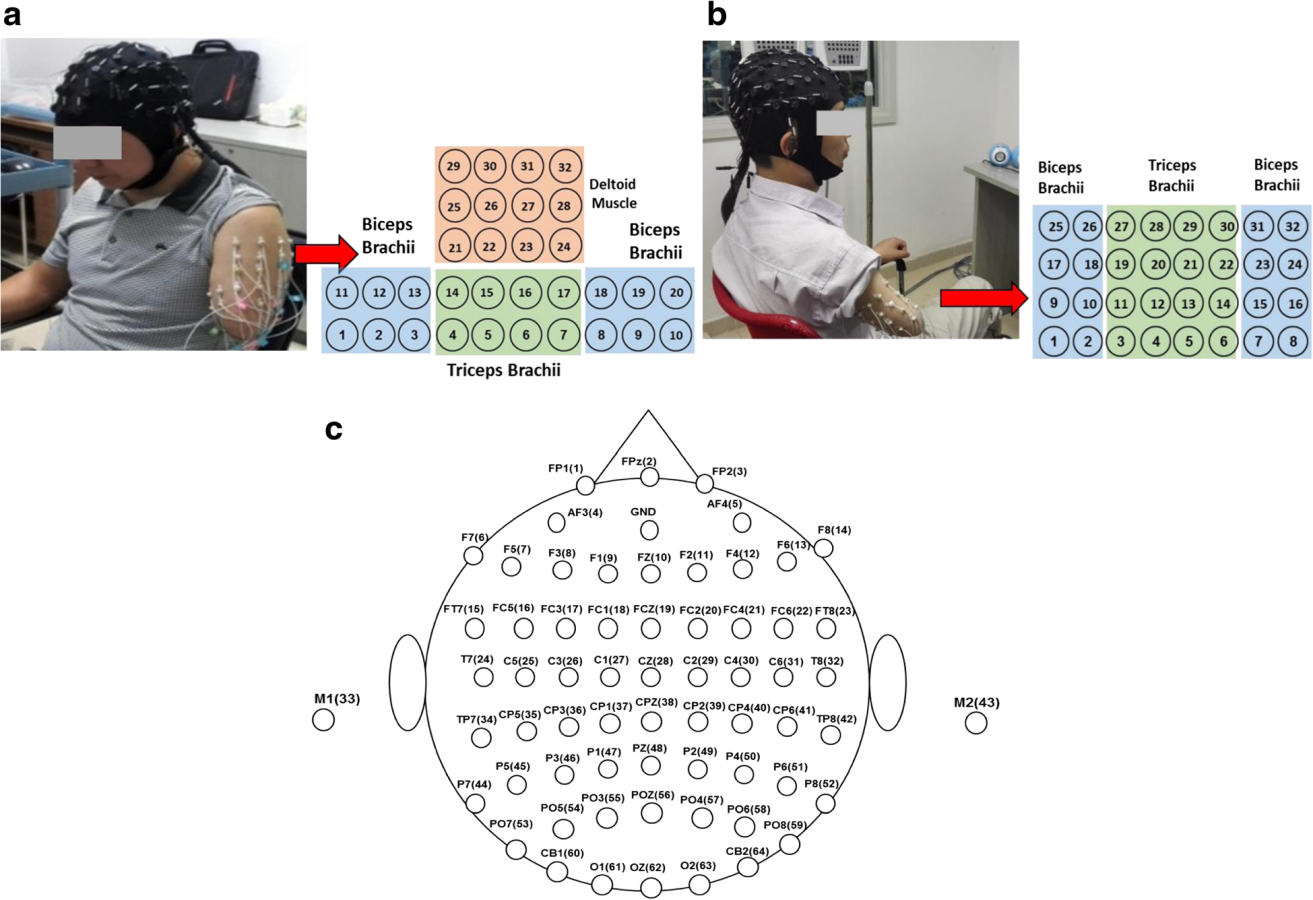
Electrode Configurations for sEMG and EEG recordings on subjects with different amputated statuses. **a** sEMG electrode placement on TH1; **b** sEMG electrode placement on TH3; **c** EEG electrodes

The sEMG and EEG were simultaneously recorded during the motion performance. The sEMG signals were collected with a high-density sEMG system (*REFA 128, TMS International, the Netherlands*), where 32 monopolar electrodes were placed on the skin surface of the residual arm for each subject. Based on the residual arm length, electrodes were placed in two different manners: For TH1 and TH2 with relatively shorter residual arms, totally 32 electrodes were distributed in a way shown in Fig. 2a, 20 of which were placed on the biceps brachii and triceps brachii as a matrix of 2 × 10, and the rest 12 of which were placed on the deltoid muscle as a matrix of 3 × 4; For TH3 and TH4 with relatively longer residual arms, all the 32 electrodes were systematically distributed on the biceps brachii and triceps brachii as a matrix of 4 × 8, as shown in Fig. 2b. In addition, an extra electrode was placed on the wrist of the intact arm as a reference. The EEG signals were acquired by a 64-channel EEG cap (*EasyCap, Herrsching, Germany*) integrated with a Neuroscan system (Version 4.3). The EEG recording system could automatically mark a vertical line on the EEG recordings as the onset/endpoint of the movement when a target motion picture appeared/disappeared on the screen. And the 64-channel Al-AgCl electrodes were distributed according to the *10–20 system standards* as shown in Fig. 2c, which is a well-accepted way to place scalp electrodes for EEG acquisition. For each subject, some preparation procedures such as hair cleaning, cap position adjusting, and impedance checking were performed to ensure that the impedance between the electrodes and scalp was lower than 10 kΩ before EEG recordings.

Both the sEMG and EEG acquisition systems used in the experiments have been integrated with a pre-filter by the producers [17, 18]. Thus the initial sEMG signals were firstly filtered with a band pass from 10 to 500 Hz and sampled at a rate of 1024 Hz; the EEG signals were filtered with a band pass from 0.05 to 100 Hz and sampled at a rate of 1000 Hz. Thereafter, both the sEMG and EEG data were preprocessed offline by using the *Matlab® 2010a*. Besides, the *EEGLAB* toolbox (*Free Software Foundation, Inc. 1991*) [19] was applied to process the EEG data, where the mean of baseline for each channel was removed and the EEG epochs of each motion class was extracted based on the onset-endpoint lines. For a motion class, each of its EEG epochs consisted of 5-s EEG recordings plus 120-ms EEG recordings before the onset of the movement, totally having 5120 data points. To further improve the quality of EEG recordings, the artifacts from some interferences such as eye movements and blinks in each EEG data epoch were detected based on an adaptive threshold that was equal to the 1.2 times of the mean absolute value of an epoch data [20]. If the absolute value of a data point of an EEG epoch was greater than the threshold, the amplitude of the data point was considered as artifact and would be replaced by the averaged value of the amplitudes of three nearby channels. In addition, a 50 Hz notch filter was used to remove the power-line noise for both sEMG and EEG recordings.

### Channel combination and motion classification

In this work, the single-signal methods with only either sEMG or EEG, and the dual-signal methods with a combination of both sEMG and EEG, were tested and compared. For the single-signal methods, three channel arrangements were specified:

1. *32-ch sEMG*, where only 32 sEMG electrodes were used;
2. *64-ch EEG*, where only 64 EEG electrodes were used;
3. *32-ch EEG*, where the first 32 of 64 EEG electrodes were used, locating on the frontal, temporal, and central lobes of cerebral cortex that are associated with thinking and motion functions [21, 22], as shown in Fig. 2c.

For the dual-signal methods, two channel combination strategies were designed:

1. *Strategy I (S-I)*, where 32 sEMG electrodes and 64 EEG electrodes were used, leading to a total of 96 channels of input, i.e. *32-ch sEMG* + *64-ch EEG*;
2. *Strategy II (S-II)*, where 32 sEMG electrodes and the first 32 of 64 EEG electrodes were used, leading to a total of 64 channels of input, i.e. *32-ch sEMG* + *32-ch EEG*.

**Fig. 3:**
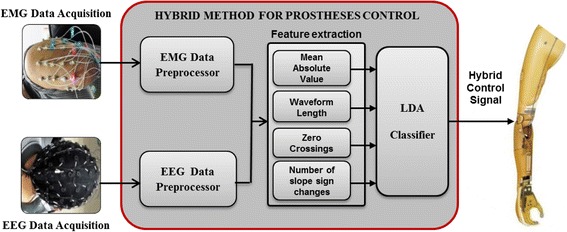
Schematic procedure for prosthesis control based on sEMG and EEG

Figure 3 schematically shows the general procedure from signal acquisition to motion classification. The preprocessed signals were segmented into a series of analysis windows with a length of 150 ms and an increment of 100 ms (with 50 ms overlapping). For each analysis window, four time-domain (TD) features of mean absolute value (MAV), waveform length (WL), zero crossings (ZC), and number of slope sign changes (SSC) were extracted [23–30]. The linear discriminant analysis (LDA) classifier [25, 27, 31, 32] was used for motion classification. The classification performance of the classifier was tested with a 5-fold cross validation method and evaluated by classification accuracy that is defined as:

1 $$ Classification\  accuracy = \frac{number\  of\  correctly\  classified\  samples}{total\  number\  of\  tested\  samples}\times 100\% $$

### Channel selection

The proposed hybrid method combining sEMG and EEG was additionally optimized by decreasing the channel number with the sequential forward selection (SFS) algorithm [31, 33, 34]. The SFS algorithm iteratively adds the most contributive electrode channel x*, which is determined by the classification accuracy (*Acc*), into the optimal channel set *S*_k_ *(k* ∈ {0, 1, ⋯, *q*}*)* as the following:

2 $$ Acc\left({S}_{k\hbox{-} 1}+{x}^{\ast}\right)=\underset{j\in \left\{1,2,\cdots, q-k\right\}}{ \max }Acc\left({S}_{k\hbox{-} 1}+{x}_j\right) $$

3 $$ {S}_k={S}_{k\hbox{-} 1}+{x}^{*} $$

4 $$ {S}_0=\varnothing $$

where *q* is the total number of channels, *k* is the number of selected channels. Suppose that *k*-1 channels have been selected into the optimal channel set *S*_k-1_ by the (*k*-1)^th^ iteration, in order to select the *k* ^th^ optimal channel x* from the set {*X*-*S*_k-1_}, each channel within the set {*X*-*S*_k-1_} was individually combined to the selected optimal channels *X*_k-1_ for motion classification. The channel which achieved the highest classification accuracy was selected as the *k* ^th^ optimal channel, and thereafter added into the *X*_k-1_ for next iteration to obtain a new optimal channel set *X*_k-1_. The iteration procedure was repeated until *k* increased to the desired level. In this work, 10 optimal sEMG channels were firstly pre-selected from the total 32 sEMG channels by using the SFS algorithm, and then three optimized dual-signal methods combining sEMG and EEG were adopted as follows:

1. *Optimized Strategy I (oS-I)*, where 20 of the 64 EEG electrodes were selected by the SFS algorithm according to Eqs. (2) ~ (4), and then combined with the pre-selected 10 optimal sEMG electrodes, achieving a total number of 30 channels that was comparable in channel number to the single-signal method of *32-ch sEMG*;
2. *Optimized Strategy II (oS-II)*, where the pre-selected 10 optimal sEMG electrodes were used as the initial channel set *S*_0_ in Eq. (4), based on which 20 EEG electrodes were selected by using Eqs. (2) and (3), and then combined with the pre-selected 10 optimal sEMG electrodes, achieving a total number of 30 channels;
3. *Optimized Strategy III (oS-III)*, where the pre-selected 10 optimal sEMG electrodes were used as the initial channel set *S*_0_ in Eq. (4), based on which only 10 EEG electrodes were selected by using Eqs. (2) and (3), and then combined with the pre-selected 10 optimal sEMG electrodes, achieving a total number of 20 channels.

## Results

Table 2 shows the classification performances for the single-signal methods of *32-ch sEMG*, *64-ch EEG*, and *32-ch EEG*, where the average classification accuracies of 77.0, 75.1 and 62.9% were achieved, respectively. Table 3 and 4 shows the classification performances of the dual-signal methods of *S-I* (*32-ch sEMG* + *64-ch EEG*) and *S-II (32-ch sEMG* + *32-ch EEG*), respectively. By *S-I*, the classification accuracy for each of the five motion classes was higher than 89%, obtaining an average value of 91.7%, and by *S-II*, the classification accuracies for different motion classes ranged from 83.9 to 94.1%, resulting in an average of 87.5%. It is clear that the dual-signal methods show much better performances than the single-signal methods both on average and for any specific motion class.

**Table 2:** Classification accuracies (%) for different single-signal methods over all the subjects

	Hand Motions		Wrist Motions		NM	Mean ± Std.
	HC	HO	WP	WS		
*32-ch sEMG*	75.8 ± 7.3	68.9 ± 5.9	78.0 ± 11.4	75.1 ± 9.9	87.2 ± 14.8	77.0 ± 6.8
*64-ch EEG*	73.9 ± 8.4	72.0 ± 4.8	71.0 ± 11.6	71.9 ± 11.0	86.7 ± 6.3	75.1 ± 6.5
*32-ch EEG*	60.7 ± 5.7	60.7 ± 6.8	56.7 ± 7.6	57.0 ± 10.9	79.3 ± 13.4	62.9 ± 9.2

**Table 3:** Classification accuracies (%) for the dual-signal method of *S-I*

	Hand Motions		Wrist Motions		NM	Mean ± Std.
	HC	HO	WP	WS		
TH 1	95.1	93.1	95.3	92.5	89.9	93.2 ± 2.2
TH 2	95.5	88.8	92.3	89.6	97.6	92.8 ± 3.8
TH 3	93.5	89.9	94.0	94.7	98.9	94.2 ± 3.2
TH 4	84.8	85.7	82.4	82.3	97.2	86.5 ± 6.2
Mean ± Std.	92.2 ± 5.0	89.4 ± 3.1	91.0 ± 5.9	89.8 ± 5.4	95.9 ± 4.1	91.7 ± 3.5

**Table 4:** Classification accuracies (%) for the dual-signal method of *S-II*

	Hand Motions		Wrist Motions		NM	Mean ± Std.
	HC	HO	WP	WS		
TH 1	87.4	88.2	89.8	88.4	82.6	87.3 ± 2.8
TH 2	93.8	85.0	91.2	82.4	98.1	90.1 ± 6.4
TH 3	81.8	82.2	91.3	91.6	98.8	89.1 ± 7.2
TH 4	82.4	80.0	76.9	80.9	96.8	83.4 ± 7.8
Mean ± Std.	86.4 ± 5.6	83.9 ± 3.5	87.3 ± 7.0	85.8 ± 5.0	94.1 ± 7.7	87.5 ± 3.0

**Fig. 4:**
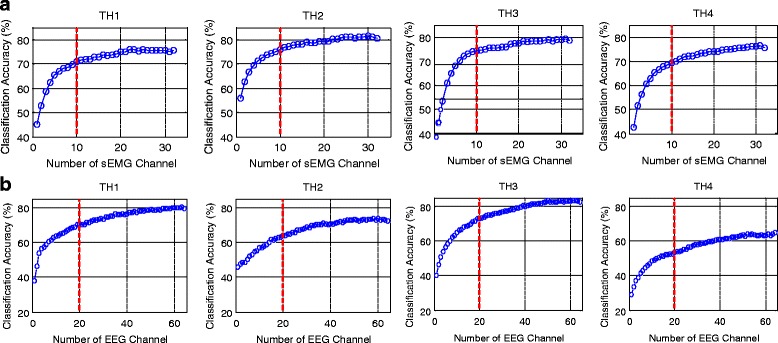
Dependence of classification performance on channel number for different subjects for **a** sEMG channels **b** EEG channels

**Fig. 5:**
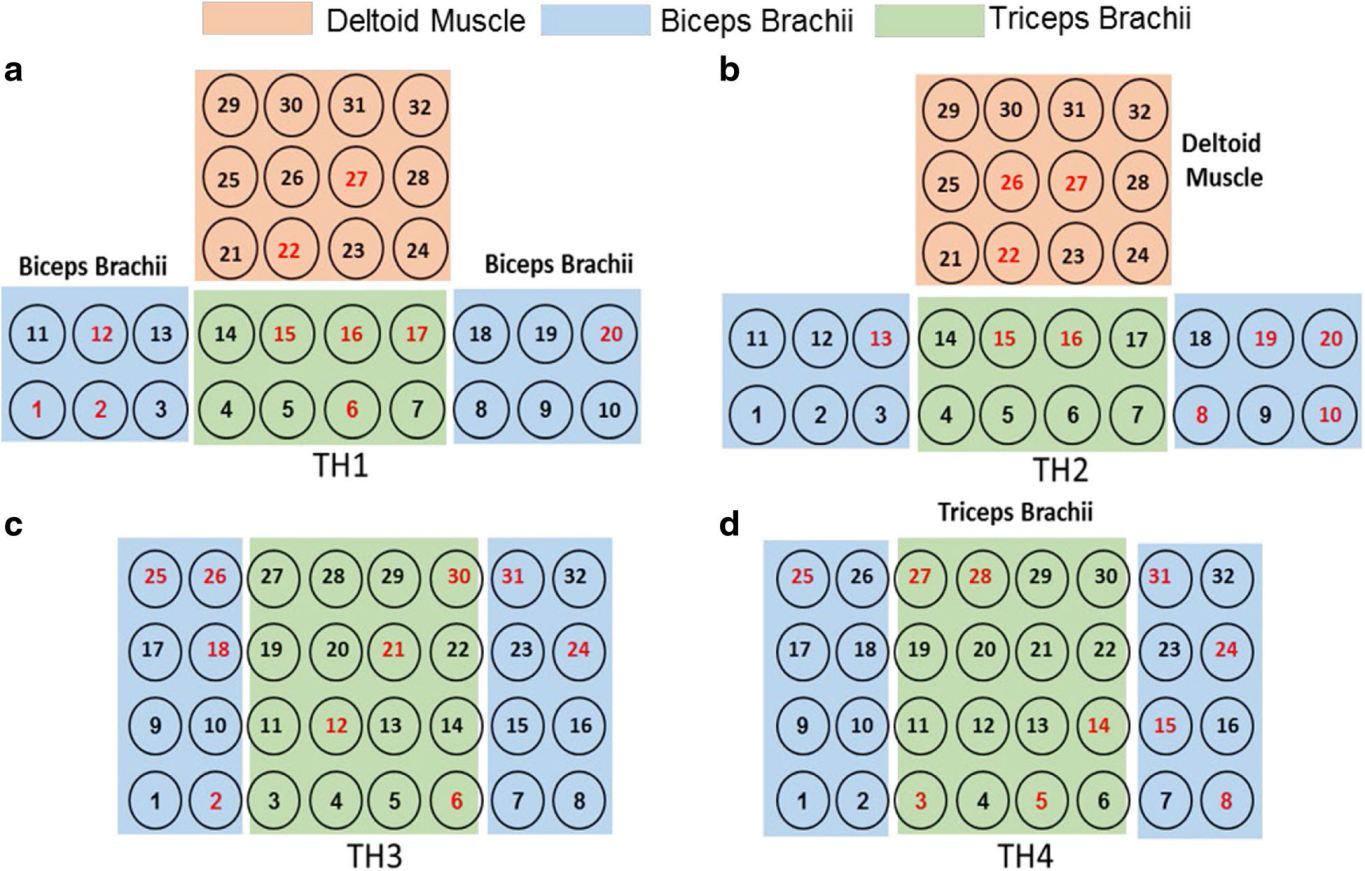
Locations of the pre-selected 10 optimal sEMG channels by using the SFS algorithm for the four subjects (**a** TH1,**b** TH2, **c** TH3, **d** TH4), as marked in *red* color

In order to achieve a satisfactory classification performance with a limited channel number that would be more practical for applications, the dual-signal methods were optimized by decreasing the channel number with the SFS algorithm. Figure 4 illustrates the dependence of classification performance on channel numbers obtained by SFS for each subject. It can be seen that the average classification accuracy increased significantly with the increase of channel number up to around 10 sEMG or 20 EEG channels, and the increase rate gradually became lower if more channels were further added. Therefore, the first 10 sEMG channels presented in Fig. 4a were pre-selected as the optimal sEMG channels for each individual subject. As marked in Fig. 5, the positions of these optimal sEMG channels varied among different subjects: for TH1 and TH2, only few channels (#22, 26, and 27) were located on the deltoid muscle, and the most were on the biceps brachii and triceps brachii; for TH3 and TH4, more optimal sEMG channels were located on the outer part of the electrode array.

Thereafter, the optimized strategy *oS-I *was implemented, where the first 20 EEG channels presented in Fig. 4b were selected by SFS and combined with the pre-selected 10 optimal sEMG channels marked in Fig. 5. Table 5 shows the classification performance of the optimized dual-signal method of *oS-I*, where the classification accuracy for each motion class was above 81.5% and the average value was 84.6%.

**Table 5:** Classification accuracies (%) for the optimized dual-signal method of *oS-I*

	Hand Motions		Wrist Motions		NM	Mean ± Std.
	HC	HO	WP	WS		
TH 1	88.0	86.4	90.2	84.0	79.1	85.5 ± 4.3
TH 2	88.5	80.9	83.7	81.9	96.8	86.4 ± 6.5
TH 3	85.3	82.0	89.0	88.2	97.9	88.5 ± 5.9
TH 4	74.8	76.7	69.0	75.2	94.4	78.0 ± 9.6
Mean ± Std.	84.2 ± 6.4	81.5 ± 4.0	83.0 ± 9.7	82.3 ± 5.4	92.1 ± 8.8	84.6 ± 4.6

**Fig. 6:**
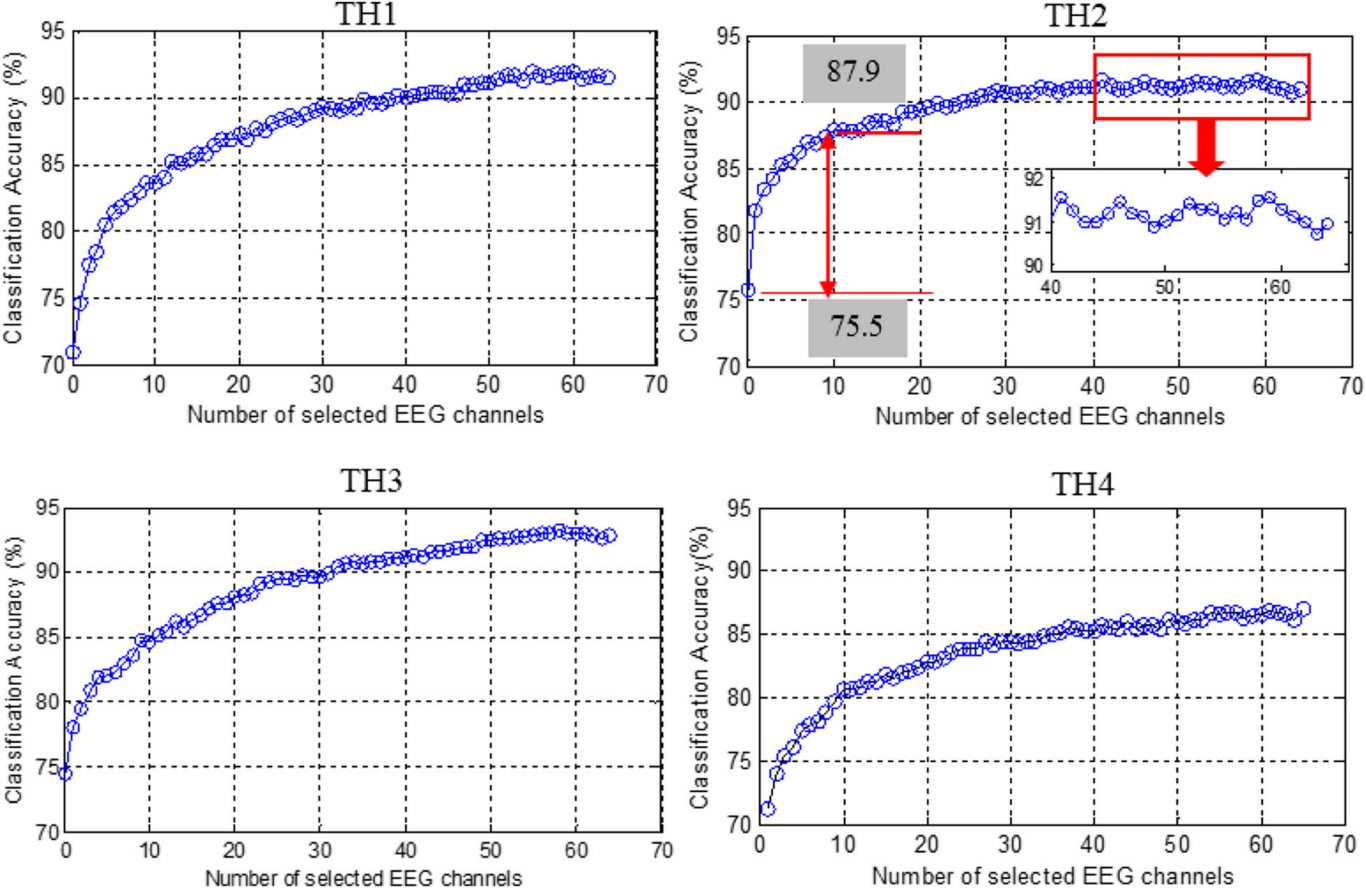
Dependence of classification performance on EEG channel number for different subjects based on the pre-selected 10 optimal sEMG channels as shown in Fig. 5

To obtain the optimized strategy *oS-II*, the pre-selected 10 optimal sEMG channels marked in Fig. 5 were initialized as the S0 in Eq. (4), based on which a channel selection analysis from the total 64 EEG channels were performed according to Eq. (3). Figure 6 shows the dependence of classification performance on EEG channel numbers obtained by SFS for each subject. As can be seen, the classification accuracies increased obviously with the increase of EEG channel number, but the increase rate slowed down if more channels were added. Especially for TH2, when the first 10 optimal EEG channels were combined to the pre-selected 10 optimal sEMG channels, the classification accuracy increased greatly from 75.5 to 87.9%; the increase slowed down if more EEG channels were added, and there was even a slight decrease if more than 40 EEG channels were used. Thus, in the optimized strategy *oS-II*, the first 20 EEG channels presented in Fig. 6 were used and combined with the pre-selected 10 optimal sEMG channels. Table 6 shows the corresponding classification performance for *oS-II*, where the mean classification accuracies for all motion classes were above 83% and the overall average value was 87.0 ± 2.7%.

**Table 6:** Classification accuracies (%) for the optimized dual-signal method of *oS-II*

	Hand Motions		Wrist Motions		NM	Average ± Std.
	HC	HO	WP	WS		
TH 1	89.7	85.7	91.8	85.1	84.5	87.4 ± 3.2
TH 2	90.4	85.1	88.4	86.2	96.7	89.4 ± 4.6
TH 3	85.1	82.9	87.5	87.0	98.0	88.1 ± 5.8
TH 4	77.0	80.1	81.7	80.5	96.5	83.2 ± 7.7
Mean ± Std.	85.6 ± 6.2	83.5 ± 2.5	87.4 ± 4.2	84.7 ± 2.9	93.9 ± 6.3	87.0 ± 2.7

**Fig. 7:**
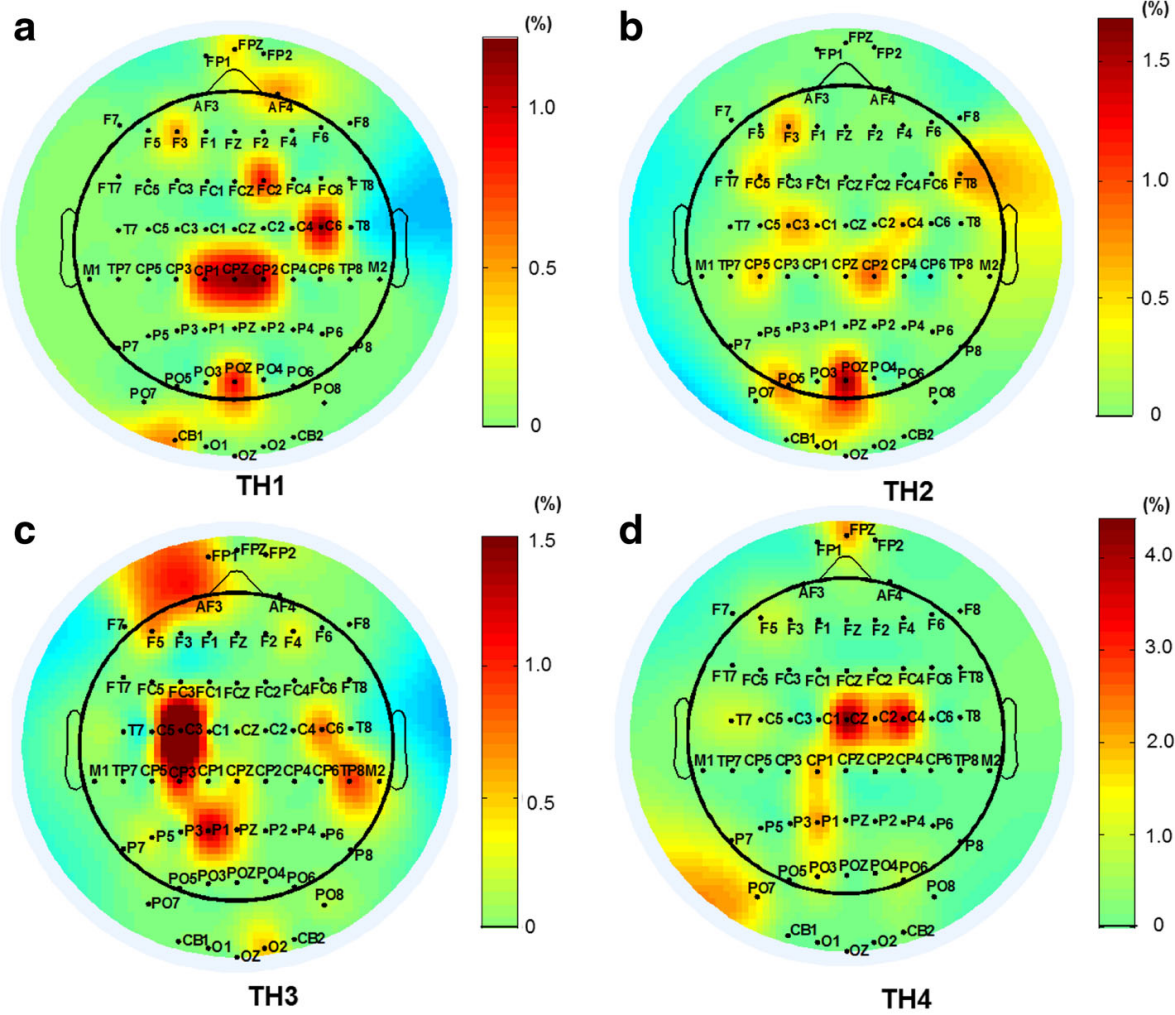
Increase rates (%) of classification accuracy by the 10 optimal EEG channels selected in the *oS-III* for the four subjects (**a** TH1, **b** TH2, **c** TH3, **d** TH4)

To further decrease the channel numbers, in the optimized strategy *oS-III*, only the first 10 EEG channels presented in Fig. 6 were selected. Figure 7 shows the increase rates of classification accuracy of the 10 optimal EEG channels used in *oS-III*. For TH1 and TH2 who had left arm amputation, the optimal channels of C6 and CP2 located on the sensorimotor cortex area of the right brain had a higher increase rate in classification accuracy than the channels on the left brain. Moreover, it was found that the EEG channel of POZ which located on the visual cortex area contributed greatly in the classification accuracy increase for both TH1 and TH2. For TH3 and TH4 who had right arm amputation, the optimal EEG channels of C3, CP3, and CZ were located on the motor cortex areas of the left brain, respectively. Tables 7 shows the classification performances for *oS-III*, where the mean classification accuracies for different motion classes were all above 78% and the overall average was 84.2 ± 3.0%.

**Table 7:** Classification accuracies (%) for the optimized dual-signal method of *oS-III*

	Hand Motions		Wrist Motions		NM	Average ± Std.
	HC	HO	WP	WS		
TH 1	86.5	79.1	91.1	81.2	80.4	83.7 ± 5.0
TH 2	89.8	81.6	86.1	84.0	98.0	87.9 ± 6.4
TH 3	79.4	76.3	86.6	85.9	95.3	84.7 ± 7.3
TH 4	74.7	77.4	75.1	80.5	95.7	80.7 ± 8.7
Mean ± Std.	82.6 ± 6.8	78.6 ± 2.3	84.7 ± 6.8	82.9 ± 2.5	92.4 ± 8.1	84.2 ± 3.0

**Fig. 8:**
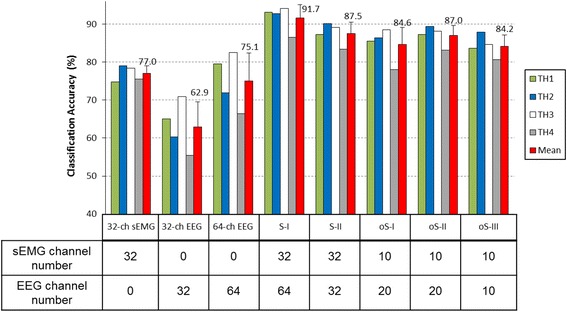
Summary of the classification performances of all the methods studied in this work

Figure 8 summarizes the classification performances of all the methods investigated in this study. Compared with the single-signal methods, the dual-signal methods could achieve higher classification accuracies. For the dual-signal method of *S-I* with 96 channels (*32-ch sEMG + 64-ch EEG*), the classification accuracy for each subject was over 85% and the average value was 91.7%, which was about 14.7% higher than that for the single-signal method of *32-ch sEMG*. By using the optimized dual-signal methods of *oS-I*, *oS-II* and *oS-III*, the total channel number was decreased to 30 or 20; however, the average classification accuracies were still about 7.6, 10.0 and 7.2% higher than those of the single-signal method with *32-ch* sEMG, respectively. Especially in *oS-III*, where only limited channel number of 10-ch sEMG and 10-ch EEG were used, the average classification accuracies of 84.2% was achieved over all the subjects.

## Discussion

Combining multiple physiological signals is a possible approach to acquire abundant neural information for prosthesis control, where the motion intentions of above-elbow amputees could be decoded more accurately. To improve the control performances of multifunctional myoelectric prostheses, a hybrid classification scheme using EEG signals as an additional augment to sEMG signals for hand and wrist motion identification is studied in this work.

With the single-signal methods that used sEMG recordings or EEG recordings only for motion classification, the *32-ch sEMG* input obtains an average classification accuracy of 77.0%, which is 1.9% higher than the *64-ch EEG* input and 14.1% higher than the *32-ch EEG* input. Thus it might be concluded that the sEMG signals usually outperform the EEG signals in motion classification since the sEMG signals generated by muscle contraction would have a more direct correlation with motions and a higher signal-to-noise ratio in comparison to EEG signals [12]. However, the sEMG is sometimes limited for some motion classes, e.g. the classification accuracy for HO in this work was only 68.9%, which was far from satisfaction for application. This is also confirmed by the subjects’ feedback that HO was the most difficult to perform in the tests. The possible reason would be that when a transhumeral amputee does a hand motion using his lost arm, his residual upper-limb muscles would generate quite weak sEMG signals since these muscles are not electro-physiologically associated with hand movements [21, 34–36].

On the other side, the EEG signals produced by mental activities are less dependent on, or even independent of, the amputation conditions. Therefore, EEG signals would be an effective auxiliary signal for sEMG to improve the pattern recognition performances. In this study, sEMG and EEG signals were firstly combined in two different designs (*S-I* and *S-II*) for motion classification. As shown in Table 3, by the dual-signal method of *S-I* (*32-ch sEMG + 64-ch EEG*), an average classification accuracy of 91.7% over all the motion classes was achieved. Especially, the classification accuracy for HO was 89.4%, which was 20.5% higher than that by the single-signal method of *32-ch sEMG* method. This result has clearly demonstrated that combining some non-EMG signals, such as EEG, to sEMG signals could significantly improve the classification performance for some forearm motions on transhumeral amputees. Different from *S-I*, the dual-signal method of *S-II* (*32-ch sEMG + 32-ch EEG*) used only the first 32 of total 64 EEG channels located on the cerebral cortex area, which is associated with the thinking and motion functions [20, 21]. Compared with the *S-I*, by which the classification accuracy was improved by 14.7% (from 77.0 to 91.7%), the *S-II* could still improve the classification accuracy by 10.5% (from 77.0 to 87.5%), meaning that the first 32 EEG channels contributed the most part of performance improvement.

There is generally a direct proportion between the motion classification performance and channel number, as demonstrated by the results of this work (Figs. 4 and 6) and some previous studies [6, 33]. However, a large number of electrodes are not feasible in actual applications partly due to the users’ comfort, computation complexity, prostheses’ weight and cost, and etc., which would eventually affect the acceptability of prostheses [34, 37]. Thus, a balance between the electrode number and control performance is very essential to realize applicable prosthetic systems. The SFS algorithm is a commonly used channel-selection method [33, 34] and it was adopted in this study to optimize the proposed hybrid strategy. According to the channel analysis shown in Fig. 4, for both sEMG and EEG signals, the classification accuracies increased significantly with the increase of channel number until about 10, but the increase slowed down if more channels were further added, which means that the last set of signal channels may not provide much effective information for motion classification [38]. In this work, three optimized strategies of *oS-1*, *oS-II*, and *oS-III* were applied, which decreased the channel number largely at the expense of a slight decrease of classification accuracy compared with *S-I* and *S-II*. Besides, it was observed from Fig. 6 that although *oS-I* and *oS-II* had the same channel number (both with *10-ch sEMG + 20-ch EEG*), the average classification accuracy of *oS-I* was 2.4% lower than that of *oS-II*. A possible reason is that in *oS-I*, the optimal 10-ch sEMG and 20-ch EEG were selected by using the SFS algorithm from the 32 sEMG and 64 EEG channels, respectively, and therefore they might not be the best matching group for motion classification, which is consistent with the conclusion by Cover [39]. Contrarily, in *oS-II*, the 20 EEG channels were picked out based on the pre-selected 10 optimal sEMG channels, and thus the matching degree between the sEMG and EEG is much higher. In this work, the optimized method *oS-II* (*10-ch sEMG + 20-ch EEG*) could obtain an average classification accuracy of 87.0%, which is very comparable to *S-II* (*32-ch sEMG + 32-ch EEG*) of 87.5%, but its channel number was less than half of *S-II*. By *oS-III*, where totally only 20 channels (*10-ch sEMG + 10-ch EEG*) were used, the classification accuracy for each subject was above 80.0% with an overall average of 84.2%, which is still about 9.1 and 7.2% higher than those of the single-signal methods of *64-ch EEG* and *32-ch sEMG*, respectively. These results demonstrated that the proposed strategies have been properly optimized by the SFS algorithm, and the *oS-III* might be quite desirable for the classification of hand and wrist motions for above-elbow amputees. If higher classification accuracy is desired, the *oS-II* could be applied. Additionally, the performances of the proposed strategies from TH4 were lower than those for other subjects. A possible reason might be that TH4 has longer and thicker hairs, which leaded to higher impedance between the scalp and the EEG electrodes, resulting in the noisier EEG recordings in comparison to others. So the quality of EEG recordings would be very important for achieving high classification accuracy.

From Fig. 5 it can be observed that the optimal sEMG channels varied among different subjects, which is due to their different status of arm amputations such as different length of remaining arms, different circumstance of arms, and different amputated durations. However, except for few electrodes located on the deltoid muscle for TH1 and TH2, most of the optimal sEMG channels were located on the muscle belly of biceps brachii and triceps brachii of the four subjects. From Fig. 7 it can be seen that the optimal EEG channels were also different in the four subjects, but most of them were located on the contralateral motor cortex. Moreover, for both TH1 and TH2, the EEG channel of POZ located on the visual cortex area contributed greatly in the classification accuracy increase. This is reasonable because the subjects performed motions by looking at the instruction pictures and thus the visual cortex area were easily activated, and TH1 and TH2 who had shorter residual arms might need activate more extra brain function areas such as visual cortex area to complete the motions.

In this study, the results were obtained based on offline analysis, and the performances of the proposed hybrid methods were only evaluated by the classification accuracy. In the future, the efficiencies of hybrid methods, especially the one with optimized channel sets, would be investigated in real-time environment and assessed by more measures such as motion selection time, motion completion time, and motion completion rate [7, 40]. Note that the 20 channels (10-ch sEMG + 10-ch EEG) were finally selected for motion classification, but they may be still too many for the practical applications of the proposed method. For the real-time applications such as myoelectric prosthetic systems, the number of electrodes should be as few as possible to reduce the complexity of the systems and make them more practical, but reducing the electrode number may decreasing the classification accuracy of movements. Thus there is a tradeoff between channel number and classification performance that should be considered in the practical applications. In the future studies, some approaches such as long term training, concentric bipolar electrode, and different classification algorithms would be used to further reduce the channel number and improve the classification performance. In addition, another limitation of the current study is that the proposed method involved EEG recording, which would not be feasible outside the controlled environment of the lab since it is impossible to wear and carry on a commercially available EEG systems such as the NeuroScan in the practical applications. With rapid development of electronics and microprocessors, it is possible now to develop a miniature, portable, and wireless EEG system for the applications of the proposed method in the prosthetic control such as the microEEG device developed by Omurtag et al. [41].

## Conclusion

In this study, hybrid classification methods based on the combination of EEG and sEMG signals were proposed for the classification of hand and wrist motions for above-elbow amputees. By comparing the dual-signal methods with single-signal methods, a significant improvement in classification accuracy was achieved on all the subjects. By using the SFS algorithm, two optimal channel sets of *10-ch sEMG + 20-ch EEG* and *10-ch sEMG + 10-ch EEG* were obtained with desirable classification accuracies of 87.0 ± 2.7% and 84.2 ± 3.0%, respectively. The results of this study might be helpful in realizing the control of multifunctional myoelectric prostheses for above-elbow amputees.

## Abbreviations

Acc: Accuracy
BCI: Brain computer interfaces
EEG: Electroencephalography
EMG-PR: EMG pattern recognition
HC: Hand close
HO: Hand open
LDA: Linear discriminant analysis
NIR: Near-infrared
NM: No movement
sEMG: Surface electromyography
SFS: Sequential forward selection
SSVEPs: Steady-state visual evoked potentials
TH: Transhumeral
WP: Wrist pronation
WS: Wrist supination
